# A Treasure of Bioactive Compounds from the Deep Sea

**DOI:** 10.3390/biomedicines9111556

**Published:** 2021-10-28

**Authors:** Assunta Saide, Chiara Lauritano, Adrianna Ianora

**Affiliations:** Marine Biotechnology Department, Stazione Zoologica Anton Dohrn, Villa Comunale, 80121 Napoli, Italy; assunta.saide@szn.it (A.S.); adrianna.ianora@szn.it (A.I.)

**Keywords:** marine organisms, bioactivities, human health, drug discovery, marine biotechnology

## Abstract

The deep-sea environment is a unique, challenging extreme habitat where species have had to adapt to the absence of light, low levels of oxygen, high pressure and little food. In order to survive such harsh conditions, these organisms have evolved different biochemical and physiological features that often have no other equivalent in terrestrial habitats. Recent analyses have highlighted how the deep sea is one of the most diverse and species-rich habitats on the planet but less explored compared to more accessible sites. Because of their adaptation to this extreme environment, deep-sea species have the potential to produce novel secondary metabolites with potent biological activities. Recent advances in sampling and novel techniques in microorganism culturing and chemical isolation have promoted the discovery of bioactive agents from deep-sea organisms. However, reports of natural products derived from deep-sea species are still scarce, probably because of the difficulty in accessing deep-sea samples, sampling costs and the difficulty in culturing deep-sea organisms. In this review, we give an overview of the potential treasure represented by metabolites produced by deep marine species and their bioactivities for the treatment and prevention of various human pathologies.

## 1. Introduction

Over the past 50 years, approximately 30,000 marine natural products (MNPs) have been reported from marine flora and fauna, with less than 2% of these derived from deep-water marine organisms (https://pubs.rsc.org/marinlit, accessed on 27 August 2021 and the work in [1]). According to the literature, those considered as “deep environments” are variable, ranging from 10 m to thousands of meters. In the current study, we consider the deep sea as the part of the ocean below the 200 m depth and representing the largest and least explored biome on Earth [2]. Less than < 0.0001% of the deep ocean has been investigated so far and, contrary to past hypotheses of it being food-poor, metabolically inactive and composing a minor component of global carbon cycles, population complex spatial structures and widespread symbioses have been observed in the deep sea [3,4,5]. For many years, the difficulty to reach the bottom of the ocean has been the main issue for studying deep-sea life. Thanks to improved acoustic technology and improved access by Remotely Operated Vehicles (ROV) and submersibles, the deep-ocean environment has become more accessible, revealing the presence of biological activity in many deep-sea species [6].

The number of known marine species is ~250,000 [1] and several recent projects have helped and are helping in discovering new species by better exploring the world’s oceans, including deep and cold extreme environments (e.g., the Census of Marine Life (http://www.coml.org/index.html; accessed on 7 October 2021), EUROFLEETS2 funded Project PharmaDeep (https://www.eurofleets.eu/access/previous-calls/eurofleets2-regional-2-call-results/eurofleets2-funded-project-pharmadeep-results/; accessed on 7 October 2021) and Tara Oceans and Tara Oceans Polar Circle expeditions (https://oceans.taraexpeditions.org/en/m/about-tara/les-expeditions/tara-oceans/; accessed on 7 October 2021). These projects focused on assessing the diversity, distribution, and abundance of marine life, are revealing that the total number of marine species worldwide may reach hundreds of millions [1].

**Figure 1:**
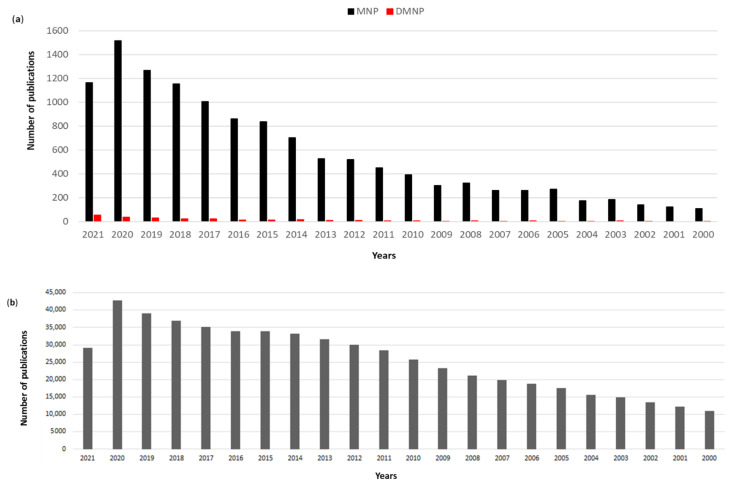
PubMed search results 2000–2021 by using as filters (**a**) the words “marine” and “natural products” for marine natural products (MNP) and “deep”, “marine” and “natural products” in “all fields” for deep marine natural products (DMNP), respectively, (**b**) the words “natural products” in “all fields”.

Searching the available literature in the PubMed database, there is a clear recent increasing trend in the number of publications in drug discovery from marine organisms and from deep-sea species (search filters used were the word “marine” and “natural products” in “all fields” and “deep”, “marine” and “natural products” in “all fields”, respectively; Figure 1). Interestingly, whereas the percentage of deep marine natural products papers was approximately 0.7–2.4% of the total publications on marine natural products until 2020, as of 2021 this percentage has increased to 4.55% denoting a very recent increasing interest in deep-sea compounds. At present, 135,828 patents related to marine natural products are available (by searching for “marine natural products patents” on https://patents.google.com/?q=marine+natural+products&type=PATENT&dups=language; accessed on 7 October 2021). Marine drug discovery is still considered a “new” research field as shown by the huge difference in the number of publications (Figure 1a,b). In 2020, only 4% of the publications on natural products were related to MNPs. At present, over 400,000 NPs are known and new compounds are being discovered every year [7,8], while ~30,000 (7.5% of total NP) are the number of MNPs identified to date since the first report of a biologically active MNP spongothymidine in 1950 [9].

The increasing interest in MNPs is largely due to the novel chemical structures found in marine organisms which have greater chemical novelty with respect to their terrestrial counterparts [10]. Approximately 70% of structural scaffolds identified in MNPs are only found in marine organisms [11]. The natural product drug discovery pipeline greatly depends on this novelty; many researchers are looking for novel marine scaffolds and, considering that the deep ocean has been less explored compared to the surface (less than 1000 deep MNP have been reported [1,12]), there is a higher probability to find new scaffolds in deep-sea species. In the deep-sea environment, there are a high number of single rare species, with more than half being new to science, with some taxa possessing >95% of undescribed species. In addition, many of the species are found to exclusively inhabit the deep sea, with high levels of biodiversity extending to abyssal depths of 5000 m [13]. There are also various recent papers analyzing the characterization, cloning, expression and functional characterization of enzymes from deep-sea organisms along with investigations into the microbial diversity of deep-sea sponge-associated bacteria and other deep-sea sediment-derived microbes [14,15,16].

**Figure 2:**
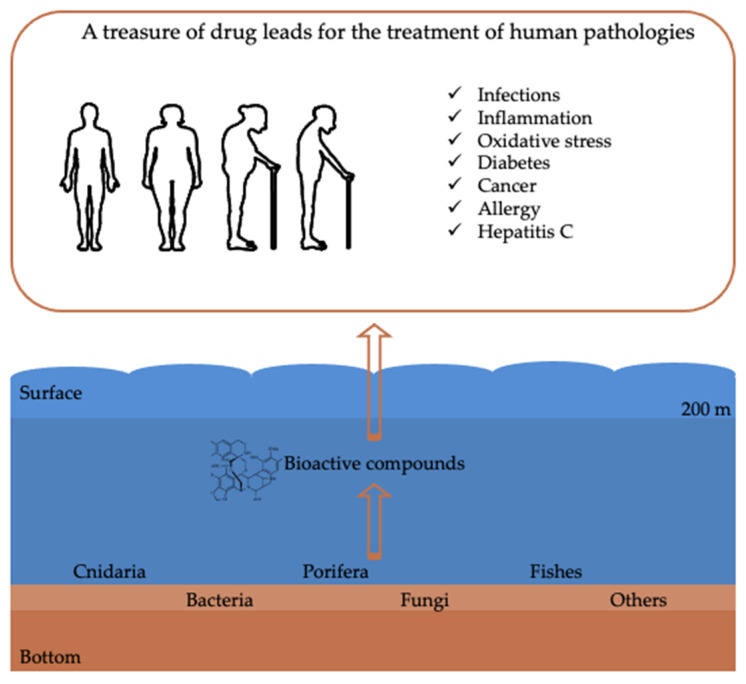
Schematic representation showing the main deep-sea marine organisms from which compounds with bioactivities useful for the treatment of human pathologies have been reported. Arrows indicate the flow from sampling of marine organisms from deep environments to the identification of bioactive compounds and possible applications for the treatment of human pathologies.

The deep sea is characterized by a pressure increase of one atmosphere (atm) for every 10 m increase in water depth, so pressure varies from 20 atm at the shelf-slope break to >1000 atm in the deepest parts of ocean trenches; temperature usually drops with increasing depth reaching values around 2 °C on the abyssal plain. The oxygen concentration in bottom waters can be much less than that of the surrounding region, or even zero, depending on the balance between the rate at which oxygen is supplied and the rate at which it is consumed. Light intensity declines exponentially with depth in the water column because incident photons are absorbed or scattered, and total darkness prevails below 250 m. Organisms inhabiting these harsh environments have developed unique strategies to adapt and survive, e.g., long lifespans, tolerance to toxic compounds at high concentrations, widespread symbiotic relationships, sound emissions, chemical signals and bioluminescence [5,17]. Their adaptation to biochemical and physiological processes may be reflected in modifications in gene regulation of primary/secondary metabolic pathways that result in the expression of novel natural products. The purpose of this review is to shed light on deep-sea metabolites and on a large number of species that produce compounds with potential activities against different human pathologies (e.g., anti-inflammatory, anticancer, antioxidant and antibacterial activities) (Figure 2). This review summarizes the state-of-the-art, and critically discusses the limits and possible advantages of drug discovery research from the deep sea.

**Figure 3:**
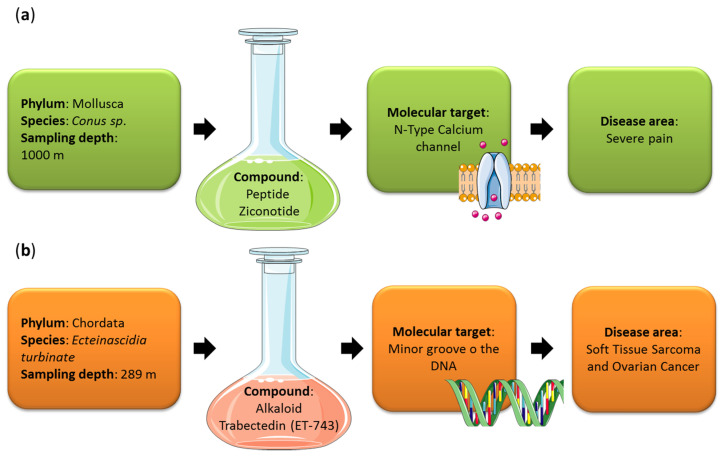
Two deep marine derived drugs are currently on the market: (**a**) Prialt^®^ and (**b**) Yondelis^®^. The figure shows species and sampling depth from which the compounds have been isolated, their molecular targets and disease areas. (**a**) Ziconotide (Prialt^®^) was derived from the venom of a marine snail (*Conus* sp.) and is used for the treatment of severe pain. (**b**) Trabectedin, ET-743 (Yondelis^®^), was derived from a marine tunicate *Ecteinascidia turbinate* and is used for the treatment of soft tissue sarcoma and ovarian cancer.

**Figure 4:**
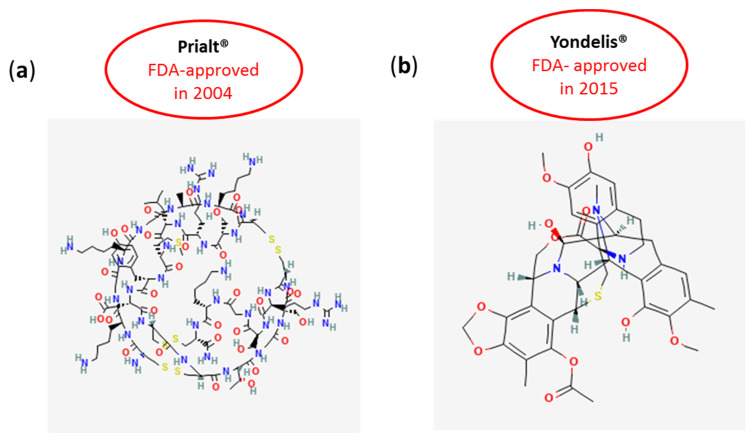
Chemical structures of (**a**) Prialt^®^ and (**b**) Yondelis^®^ FDA approved in 2004 and 2015, respectively. Chemical structures of Ziconotide (Prialt^®^; PubChem Identifier: CID 16135415) and Trabectedin, ET-743 (Yondelis^®^; PubChem Identifier: CID 108150), are from the public database PubChem (https://pubchem.ncbi.nlm.nih.gov/compound/Ziconotide#section=2D-Structure [20] and https://pubchem.ncbi.nlm.nih.gov/compound/Trabectedin#section=2D-Structure [21], both accessed on 11 October 2021).

Currently, there are fourteen (13 Food and Drug Administration (FDA)-approved drugs and one Australia drug approved in 2018) pharmaceutical products in clinical use, of which nine are used for different cancer treatments (Cytosar-U^®^, Halaven^®^, Adcetris^®^, Yondelis^®^, Aplidin^®^, Polivy^TM^, PADCEV^TM^, Zepzelca^TM^ and Blenrep^TM^), three for hypertriglyceridemia (Lovaza^®^, Vascepa^®^ and Epanova^®^), one for severe chronic pain (Prialt^®^) and one as antiviral (Vira-A^®^), against herpes simplex virus (https://www.midwestern.edu/departments/marinepharmacology/clinical-pipeline, accessed on 12 October 2021). Their mechanisms of action and, in particular, their molecular targets are very variable. For the anticancer drugs, the molecular targets are DNA polymerase, microtubules, CD30/microtubules, minor groove of DNA, translation elongation factor 1A2 (eEF1A2), CD76b/microtubules, Nectin-4, RNA Polymerase II and B-cell maturation antigen (BCMA), for Cytosar-U^®^, Halaven^®^, Adcetris^®^, Yondelis^®^, Aplidin^®^, Polivy^TM^, PADCEV^TM^, Zepzelca^TM^ and Blenrep^TM^, respectively. Regarding Lovaza^®^, Vascepa^®^ and Epanova^®^, their molecular targets are tryglyceride-synthesizing enzymes. For Prialt^®^, the molecular target is a N-Type Calcium channel, while for Vira-A^®^ a viral DNA polymerase (https://www.midwestern.edu/departments/marinepharmacology/clinical-pipeline, accessed on 12 October 2021). Another seven marine-derived drugs are in phase I clinical trials (seven for cancer treatment and one for HIV prevention), twelve are in phase II (10 for cancer treatment, one specific for Alzheimer’s disease and one for schizophrenia, Alzheimer’s disease, attention deficit hyperactivity disorder, endotoxemia, sepsis and vagal activity) and four are in phase III (three against cancer and one for chronic pain). These compounds have been identified mainly from invertebrates, such as sponges, mollusks, bryozoans and ascidians, or microorganisms, mainly cyanobacteria. Cancer treatment is the most frequent field of application, maybe because these compounds have defensive roles in the natural environments. One of these derives from the deep sea (Figure 3). Ziconotide (Prialt^®^) is a synthetic calcium channel-binding conotoxin isolated from the sea snail *Conus magus* living at depths greater than 1000 m, used for the treatment of severe pain (it was approved by the FDA in 2004) [18]. In 2015, an anticancer agent isolated from the tunicate *Ecteinascidia turbinate*, sampled at 289 m depth, was FDA approved as trabectedin (Yondelis ^®^) for the treatment of soft tissue sarcomas and ovarian cancer [19]. Chemical structures of Prialt^®^ and Yondelis ^®^ are reported in Figure 4. Sampling depth of the species from which MNPs have been isolated is not always available and it is difficult to establish how many other drugs come from the deep sea. The pre-clinical pipeline continues to supply several novel MNPs every year potentially enriching the clinical pipeline with valuable new drugs. Below, we discuss the current state of art of deep-sea species which showed bioactivities useful for the treatment of human pathologies.

## 2. Deep-Sea Species with Biological Activities

### 2.1. Bacteria

Marine bacteria from deep-sea sediments have shown to be a rich source of secondary metabolites with novel structures and excellent biological activities, including antiproliferative, antimicrobial and antimalarial activities [22]. In this paragraph, we report twenty-four bacteria that have been isolated from deep-sea sediments known to produce bioactive compounds (Table 1). The genus *Salinispora* is an obligate seawater-requiring marine actinomycete [23] and includes *Salinispora tropica*, *Salinispora arenicola* and *Salinispora pacifica. Salinispora tropica* is found in tropical and subtropical marine sediments at depths up to 1100 m [23]. *S. tropica* produces the potent proteasome inhibitor salinosporamide-A, characterized by a densely functionalized γ-lactam-β-lactone bicyclic core that is responsible for its irreversible binding to the β subunit of the 20S proteasome. This compound displayed potent and highly selective activity in the National Cancer Institute’s (NCI) 60-cell line panel with a mean GI_50_ value (the concentration required to achieve 50% growth inhibition) of less than 10 nM and a greater than 4 log LC_50_ differential between resistant and susceptible cell lines. Better inhibition (LC_50_ < 10 nM) was observed against NCI-H226 non-small cell lung cancer, SF-539 CNS, SK-MEL-28 skin melanoma and MDA-MB-435 breast cancer [24].

Nachtigall et al. [25] identified benzoxacystol 245 from the bacterium *Streptomyces* sp. strain NTK 935 (sampling depth 3814 m), that is the first benzoxazine reported to have an inhibitory activity against glycogen synthase kinase 3β (GSK-3β) and inhibited the recombinant enzyme in an in vitro enzyme activity assay with an IC_50_ value of 1.35 ± 0.15 μM (Table 1). GSK-3β is a key regulator of numerous signaling pathways and has emerged as a prominent target for the treatment of Alzheimer’s disease and type 2 diabetes. In addition, benzoxacystol 245 exhibited weak antiproliferative activity against mouse fibroblast NIH-3T3 cells, inhibiting 18% cell growth at a concentration of 50 μM.

For the family lobophorin, Braña et al. [26] isolated a new natural product designated as lobophorin K, from the marine actinobacterium *Streptomyces* sp. M-207, previously isolated from the cold-water coral *Lophelia pertusa* collected at 1800 m. This new natural product displayed cytotoxic activity against two human tumor cell lines, pancreatic carcinoma (MiaPaca-2) and MCF-7, with IC_50_ values of 34.0 ± 85.1 and 23.0 ± 8.9, respectively (Table 1).

A novel compound, named 7,13-epoxyl-macrolatin A, was isolated from *Bacillus subtilis B5* that is an inhibitor of lipopolysaccharide (LPS)-induced inflammatory mediator expression in RAW264.7 cells (macrophage cell lines) [27]. This compound was isolated from sediments collected at a depth of 3000 m in the Pacific Ocean and exhibited a more potent inhibitory effect on nitric oxide (NO) production and several inflammatory cytokines than the previously known macrolactins, such as macrolactin A and analogues. This compound also inhibited the mRNA expression of the inducible nitric oxide synthase (iNOS), interleukin-1β and interleukin-6 in LPS-stimulated RAW 264.7 cells.

In 2011, Huang et al. [28] isolated different compounds from *Marianactinospora thermotolerans* SCSIO00652 (sampling depth 3865 m), named marinacarbolines A-D, 13-N-demethyl-methylpendolmycin, methylpendolmycin-14-*O-*α-glucoside. Huang et al. demonstrated that marinacarbolines had antimalarial activity against *Plasmodium falciparum* (Table 1).

Other compounds isolated from deep bacteria showed interesting antimicrobial activities. In particular, marthiapeptide A, derived from *Marinactinospora thermotolerans* SCSIO 00652, sampled at 3865 m in the South China Sea, also showed antimicrobial activity. Zhou et al. [29] showed that this compound had antimicrobial activity against *Microccocus luteus*, *Staphilococcus aureus*, *Bacillococcus subtilis* and *Bacillococcus thuringiensis*, with minimum inhibitory concentration (MIC) values of 2, 8.4 and 2 μg/mL. Marthiapeptide A was also tested for cytotoxic activity against the human glioblastoma cell line (SF-268), the human breast adenocarcinoma cell line (MCF-7), the human lung carcinoma cell line (NCI-H460) and the human hepatocarcinoma cancer cell line (HepG2). Activity testing showed IC_50_ (concentration of drug required for 50% inhibition) values ranging from 0.38 to 0.52 μM [29]. In 2014, Song et al. [30] isolated from the deep-sea organism *Streptomyces scopuliridis* SCSIOZI 46 (sampling depth 3536 m) the compounds desotamides. In particular, desotamide B, C and D showed antimicrobial activity against *S. aureus*, *S. pneumoniae* and methicillin-resistant *Staphylococcus epidermidis* (MRSE) with a MIC value of 16, 12.5 and 32 μg/mL, respectively. Zhou et al. [31] isolated from the bacterium *Streptomyces drozdowiczii* SCSIO10141 (sampling depth 1396 m) marfomycins A, B and E, which also showed antimicrobial activity against *M. luteus* with MIC values of 0.25, 4, and 4 μg/mL, respectively. In 2011, Niu et al. [32] collected *Streptomyces* sp. SCSIO 01127 from 1350 m of the South China Sea and isolated lobophorin F that showed antimicrobial activity against *S. aureus* and *Enterococcus faecalis* with MIC values of 8 μg/mL (for both). According to Pan et al. [33], lobophorin H isolated from *Streptomyces* sp. 12A35 (sampling depth 2134 m) showed antimicrobial activity against *B. subtilis* with a MIC value of 3.13 μg/mL. Bister et al. [34] collected from the Japanese Sea the *Verrucosispora* sp. AB 18-032 (sampling depth 289 m) and isolated abyssomicin C that showed antimicrobial activity against *MRSA*, *vancomycin resistant Enterococcus faecium* and *S. aureus* with a MIC value of 4 μg/mL. Song et al. [35] collected the bacterium *Streptomyces niveus* SCSIO3406 from 3536 m of the South China Sea and isolated the compounds marfuraquinocins A, C and D. These molecules showed antimicrobial activity against *S.aureus* and MRSE shhs-E1 with a MIC value of 8 μg/mL (for both). Sarmiento-Vizcaíno et al. [36] isolated a novel natural product of the paulomycin family, designated as paulomycin G, obtained from the marine strain *Micromonospora matsumotoense* M-412, collected from Cantabrian Sea sediments at 2000 m depth. Paulomycin G showed antimicrobial activity against *E. coli* strains MB5746 and MRSA with a minimum concentration at which 90% of the isolates were inhibited (MIC_90_) values of 4.5 and 50 μg/mL, respectively [36]. Paulomycin G showed a strong cytotoxic activity against MCF-7, MiaPaca-2, and HepG2 cancer cell lines (Table 1). The *Bacillus subtilis* isolated from deep-sea sediments collected at a depth of 1000 m in the Red Sea was found to yield four novel amicoumacins, lipoamicoumacins A–D [37], and one new bacilosarcin analogue along with six known amicoumacins. Two of the known amide-containing amicoumacin and bacilosarcin analogues were found to display both significant cytotoxicity against HeLa cells and antibacterial activity against *B. subtilis*, *S. aureus* and *Laribacter hongkongensis.* Dermacozines A–G were reported from the actinobacteria *Dermacoccus abyssi* sp. nov., strains MT1.1 and MT1.2, isolated from Mariana Trench sediments collected at a depth of 10,898 m. Dermacozines F and G displayed moderate cytotoxic activity against the leukemia cell line K562 with IC_50_ values of 9 and 7 μM, respectively, while dermacozine C also exhibited high radical scavenger activity with an IC_50_ value of 8.4 μM [38]. The actinobacterium *Pseudonocardia* sp. strain (SCSIO 01299), recovered from deep-sea sediments obtained at 3258 m depth in the South China Sea, yielded three new diazaanthraquinone derivatives, pseudonocardians A–C; these compounds exhibited cytotoxic activity against three tumor cell lines SF-268 (central nervous system cancer), MCF-7 (breast cancer) and NCI-H460 (lung cancer) with IC_50_ values ranging between 0.01 and 0.21 μM. The compounds also showed antibacterial activities against *S. aureus* ATCC 29213, *Enterococcus faecalis* ATCC 29212 and *Bacillus thuringensis* SCSIO BT01, with MIC values of 1–4 μg mL^−1^ [39] and are the subject of a patent (CN102351859A). The actinobacteria *Streptomyces lusitanus*, obtained from 3370 m deep sediments collected in the South China Sea, provided five new C-glycoside angucycline metabolites, grincamycins B–F and a known angucycline antibiotic, grincamycin. Grincamycin A–E displayed in vitro cytotoxicities against the human cancer cell lines HepG2 (hepatocellular liver), SW-1990 (pancreatic), HeLa (epithelial carcinoma), NCI-H460 (lung) and MCF-7 (breast adenocarcinoma), and the mouse melanoma cell line (B16), with IC_50_ values ranging from 1.1 to 31 μM [40]. A deep-sea actinobacterium collected from *Streptomyces* sp. SCSIO 03032, recovered from a sediment sample at 3412 m depth in the Indian Ocean, produced four novel bisindole alkaloids spiroindimicins A–D, together with two known compounds, lynamicins A and D. Spiroindimicins B–D with a (5,5) spiro-ring displaying moderate cytotoxicities against several cancer cell lines [41]. A further *Streptomyces* sp. (NTK 937) recovered from an Atlantic Ocean deep-sea sediment core collected at a depth of 3814 m, yielded a new benzoxazole antibiotic, caboxamycin which exhibited inhibitory activity against Gram-positive bacteria and the enzyme phosphodiesterase as well as cytotoxic activity towards gastric adenocarcinoma (AGS), hepatocellular carcinoma (HepG2) and a breast carcinoma cell line (MCF-7) [42]. Diketopiperazines, isolated from *Nocardiopsis alba* sampled at >1000 m, showed cytotoxic activity against MCF7 and SF-268 human cancer cell lines with IC_50_ values of 4.6 and 12.7 μM, respectively [43]. In 2012, Moushumi Priya and Jayachandran [44] isolated the marine *Bacillus pumilus* MB 40 from deep sea water column (1000 m depth) near Andaman and Nicobarislands (Islands, India) that produced a bioactive lead, bis(2-ethylhexyl) phthalate (BEHP). This compound activity was examined in human erythroleukemic K562 cells using the 3-(4,5-dimethylthiazol-2-yl)-2,5-diphenyltetrazolium bromide (MTT) assay and the IC_50_ was found to be 21 μM [44]. BEHP was able to induce apoptosis involving caspase pathways, besides regulating mitochondrial enzymes.

### 2.2. Fungi

The deep-sea fungi have also been shown to produce metabolites with various possible bioactivities (such as antimicrobial, anti-inflammatory, antioxidant and anti-diabetes) and can be a potential addition to modern medicine, industry and agriculture [45]. We found twenty species of deep fungi (Table 2) which have been shown to be producers of bioactive molecules. Zhang et al. [45] investigated the diversity of fungal communities in nine different deep-sea sediments. The samples were collected at approximately 2400–4000 m depths during the South China Sea Open Cruise in August 2011. Twenty-seven fungal representatives were tested against marine pathogenic bacteria and two marine pathogenic fungi to examine their spectra of antimicrobial activity. The authors [45] found that some isolates belonging to the genera *Aspergillus* and *Penicillium* exhibited relatively high antibacterial and antifungal activity. The antimicrobial activities of fungal isolates were determined by a double-layer technique. Two marine pathogenic bacteria, *Micrococcus luteus* (ML) and *Pseudoalteromonas piscida* (PP), and two marine pathogenic fungi, *Aspergillus versicolor* (AV) and *Aspergillus sydowii* (AS), were used as the indicator microorganisms for the double-layer assay [45]. *Aspergillus fischeri* FS452 was isolated from deep-sea sludge in the Indian Ocean at 3000 m depth. Fiscpropionates A–D obtained from this fungus exhibited significant inhibitory activities against *Mycobacterium tuberculosis* protein-tyrosine phosphatase B, an important virulence factor. Fiscpropionates A and C were comparatively effective, with IC_50_ values of 5.1 and 4 μM, respectively [46]. The emerixanthones A-D isolated from the deep-sea fungus *Emericella* sp. SCSIO05240 (sampling depth at 3258) showed antibacterial activity against *Escherichia coli*, *Klebsiella pneumoniae*, *Staphilococcus aureus*, *Enterococcus faecalis*, *Acinetobacter baumannii* and *Aeromonas hydrophila* [47].

Three new phenolicbisabolane sesquiterpenes—peniciaculins A and B (1 and 2) and (7S)-(−)-10-hydroxysydonic acid (3)—as well as a new nor-bisabolane derivative 1-hydroxy-boivinianin A (4), and six known bisabolane sesquiterpenes (5−10), were isolated and identified from the culture extract of *Penicillium aculeatum* SD-321 found in the South China Sea at a depth of 2038 m [48]. Compounds 1−10 were evaluated for antimicrobial activity against two pathogenic bacteria (*Escherichia coli* and *Staphyloccocus aureus*) and eight aquatic pathogens (*Aeromonas hydrophilia*, *Edwardsiella tarda*, *Micrococcus luteus*, *Pseudomonas aeruginosa*, *Vibrio alginolyticus*, *Vibrio anguillarum*, *Vibrio harveyi*, *and Vibrio parahemolyticus*) as well as three plant-pathogenic fungi (*Alternaria brassicae*, *Colletotrichum gloeos-prioides* and *Gaeumannomyces graminis*). Compound 1 showed inhibitory activity against *M. luteus* and *V. alginolyticus* with MIC values of 1.0 and 2.0 μg/mL, while compounds 2 and 4 exhibited selective antibacterial activity against *E. tarda* and *V. harveyi*, with MIC values of 8.0 and 4.0 μg/mL respectively. Compound 6 showed significant inhibitory activity against *S. aureus* and *V. parahemolyticus* with MIC values of 0.5 μg/mL. Regarding antifungal activity, compound 1 showed inhibitory activity against *A. brassicae* with a MIC of 0.5 μg against *S. aureus* and *V. parahemolyticus* with MIC values of 0.5 μg/mL which is more potent than that of the positive control amphotericin B (MIC 64 μg/mL). Compounds 1−10 were also evaluated for lethal activity against brine shrimp (*Artemia salina*), but none of them displayed significant activity (LD_50_ > 10 μg/mL) [48].

Guo et al. [49] collected *Penicillium* sp. F23-2 in Jiaozhou Bay at 5080 m depth, and isolated Penycyclones A-E that showed antimicrobial activity against Gram-positive bacterium *Staphylococcus aureus* with a MIC value from 0.3 to 1 μg/mL [49]. *Spiromastix* sp. MCCC3A00308 was isolated from sediments collected from the South Atlantic Ocean at a depth of 2869 m. A new class of phenolic lactones, including spiromastilactones A-M, was isolated from this fungus. These compounds reveal an antiviral activity against influenza virus replication in vitro [50]. In 2017 Xiao Ling and co-workers isolated from the fungus *Simplicillium obclavatum* EIODSF 020 (sampling depth at 4571 m) different cyclic peptides; in particular, some of these showed antifungal and antiviral activity including simplicilliumtide J, verlamelins A and B. Antifungal activity was detected against *Aspergillus versicolor* and *Curvularia australiensis* with a minimum inhibitory concentration of 50 μg/mL and an antiviral activity towards HSV-1 with an IC_50_ 14.0, 16.7 and 15.6 μM/mL, respectively [51]. In addition to antibacterial, antifungal and antiviral, other bioactivities were found for fungal derived compounds from the deep sea. Fifteen new eremophilane-type sesquiterpenoids, acremeremophilanes A–O, were isolated from the fungus *Acremonium* sp. from deep-sea sediments (sampling depth of 2869 m) in the South Atlantic Ocean [52]. Acremeremophilane B (EC_50_ 8 μM) and E (EC_50_ 15 μM) showed comparatively potent inhibitory effects towards LPS-induced NO production in RAW 264.7 cells with possible anti-inflammatory activity. Another fungus was collected from sediments at the extreme depth of 5610 m in the South Atlantic Ocean, the fungus *Eutypella* sp. MCCC3A00281. Using chromatographic application of the extended metabolites led to the isolation of a total of 30 eremophilane-type sesquiterpenoids, of which 26 were identified as new compounds, namely eutyperemophilane A–Z [53]. Eutyperemophilane I and J showed comparatively potent inhibitory effects towards nitric oxide (NO) production as induced by lipopolysaccharide (LPS) in Raw 264.7 macrophage cells (IC_50_ of 8.6 and 13 μM, respectively), suggesting they could be promising anti-inflammatory agents [53]. Three dimeric nitrophenyl trans-epoxyamides, chrysamides A–C, were obtained from the deep-sea fungus *Penicillium chrysogenum* SCSIO411001 collected in the Indian Ocean at a depth of 3386 m. Chrysamide C exhibited inhibitory effects on the production of the pro-inflammatory cytokine Interleukin-17. The inhibitory rate of the production of IL-17 was 40.06% at 1.0 μM, while compounds B and C did not show any inhibitory effects at 50 μM [54].

Recently, Lu et al. [55] also isolated novel compounds having anti-inflammatory activity from deep-sea fungi (sampling depth at 2130 m), a pair of 2-benzoyl tetrahydrofuran enantiomers, named (−)-1*S*-myrothecol, (+)-1*R*-myrothecol, methoxy-myrothecol, and an azaphilone derivative, myrothin, from the culture filtrates of the deep sea-derived fungus, *Myrothecium* sp. BZO-L062 [55]. The isolated compounds showed cellular anti-inflammatory activity inhibiting nitric oxide (NO) formation in LPS-treated macrophage-like cells. Furthermore, the antioxidant activity was measured by oxygen radical absorbance capacity (ORAC) and both compounds showed antioxidant activity in the ORAC assay with EC_50_ of 1.20 and 1.41 μg/mL, respectively. Very recently, Wang et al. [56] isolated two benzodiazepine derivatives—cyclopenol and cyclopenin—from the extract of the fungal strain *Aspergillus* sp. SCSIOW2, collected from deep marine sediments in the South China Sea at a depth of 2439 m. The authors demonstrated that cyclopenol and cyclopenin inhibited the lypopolysaccharide-induced (LPS) formation of nitric oxide and the secretion of interleukin-6 (IL-6) in Raw 264.7 cells. IL-6 is a soluble mediator with a pleiotropic effect on inflammation, immune response, and hematopoiesis. In particular, cyclopenol and cyclopenin were found to inhibit the upstream signal of nuclear factor-kappa B (NF-κB) activation at 50 and 100 μM, respectively. Furthermore, these compounds also inhibited the expression of interleukin-1β, IL-6 and iNOS in mouse microglia cells, and macrophages in the brain. Cyclopenol was reported to inhibit human bladder cancer cell line BIU-87 (IC_50_ of 8.34 μM) [57] and was also shown to inhibit protein-tyrosine phosphatase 1B (PTP1B) in a dose-dependent manner with an IC_50_ value of 30 μM [58]. PTP1B catalyzes dephosphorylation of phosphotyrosine residues in the insulin receptor, inhibiting post-receptor signaling in insulin responsive tissue, and is associated with the development of type-2 diabetes [68]. Fructigenine A, echinulin, flavoglaucin and viridicatol from the deep fungi *Penicillium* spp. and *Eurotium* sp were also found able to inhibit PTP1B [58].

The fungus *Graphostroma* sp. MCC3A00421 was isolated from deep-sea hydrothermal sulfide deposits from the Atlantic Ocean at a depth of 2721 m [59]. The isolated compounds from *Graphostroma* sp. were tested for cellular anti-food allergic bioactivity in antigen and IgE-treated RBL-2H3 cells (basophilic leukemia cell lines). Reticulol, a known polyketide, effectively decreased the rates of degranulation and histamine release, with IC_50_ values of 13.5 and 13.7 μM. *Penicillum granulatum* MCCC3A00475 the extract derived from this deep (sampling depth at 2284 m) fungi showed promising antiallergic activity. This was measured for the efficiency of the RBL-2H3 cell degranulation inhibition rate using an IgE-mediated mast cell allergic reaction. Spirograterpene A showed an antiallergic effect on immunoglobulin E (IgE)-mediated rat mast RBL-2H3 cells with 18% inhibition compared to 35% inhibition for the positive control, loratadine, at the same concentration of 20 μg/mL. Conidiogenone I and conidiogenone C exhibited weak effects with inhibition of 4% and 10%, respectively, at 20 μg/mL [60]. The fungus *Penicillium chrysogenum* SCSIO 07007 was isolated from deep-sea hydrothermal vents from the Western Atlantic Ocean at a depth of ~1000 m. Chrysopyrones A and B obtained from *Penicillium chrysogenum* inhibited protein-tyrosine phosphatase-1B (PTP1B) involved in diabetes mellitus [61] with IC_50_ values of 9.32 and 27.8 μg/mL, respectively. Finally, Yao et al. isolated from the deep-sea fungus *Engyodontium album* DFFSCS021, eight new chromones, engyodontiumones A-H (1–8), and eight known polyketides (9–16). These polyketide compounds show a significant selective cytotoxicity against the human histiocytic lymphoma U937 cell line with IC_50_ of 4.9–8.8 μM. In addition, compounds 8,12, and 13 exhibit mild antibacterial activity against *Escherichia coli* and *Bacillus subtilis* [62]. Luteoalbusins A and B were isolated from the fungus *Acrostalagmus luteoalbus* SCSIO F457 originating from a deep-sea sediment sample collected at a depth of 2801 m in the South China Sea [63]. The novel compounds displayed strong cytotoxic activities against four cancer cell lines (SF-268, MCF-7, NCI-H460 and HepG2) with IC_50_ values in the range of 0.23–1.31 μM and were significantly more potent that the control cisplatin. The deep-sea fungus *Aspergillus versicolor* obtained from 800 m depth in the Pacific Ocean has furnished three new sterigmatocystin derivatives: oxisterigmatocystin A–C. These compounds were evaluated for cytotoxicity towards A549 and HL-60 cell lines, and are currently the only known compounds of this class to exhibit moderately low micromolar cytotoxicity [64]. In 2009, Li’s group reported the isolation and characterization of three new bioactive breviane spiroditerpenoids, breviones F–H, from the deep-sea sediment derived fungus *Penicillium* sp. (MCCC 3A00005) collected at a depth of 5115 m in the east Pacific [69]. Brevione I exhibited significant cytotoxicity against MCF-7 cells [65]. The fungus *Penicillium* sp. (F00120), recovered from deep-sea sediments obtained at a depth of 1300 m from the northern South China Sea, yielded the new sesquiterpene quinone, penicillium A, along with known ergosterol and ergosterol peroxide. Penicillium A moderately inhibited the in vitro proliferation of mouse melanoma (B16), human melanoma (A375) and human cervical carcinoma (Hela) cell lines [66]. Sorbicillamines A-E were isolated from the deep-sea fungus *Penicillum* sp. F23-2 and evaluated for their cytotoxicity against HeLa (human cervical cancer cell line), BEL-7402 (human hepatocellular carcinoma cell line), HEK-293 (human embryonic kidney cell line), HCT116 (human colon cancer cell line) and P388 (leukemia cell line) cell lines. IC_50_ values were > 10 μM, respectively [67].

### 2.3. Cnidaria

In Sagami Bay, between 2004 and 2009, Kawabata et al. [70] collected twelve deep-sea jellyfishes from the mesopelagic zone (depth of 200–1000 m) in order to find useful polyketides, such as molecular probes for biochemical studies and drug applications. In particular, Kawabata demonstrated that three jellyfishes from the deep-sea had biological activity (Table 3). *Aegina citrea* showed cytotoxicity against leukemia cells L-1210 with an IC_50_ value of 100 mg/mL, *Crossota rufobrunea* had hemolytic activity with an ED_50_ (effective dose for 50% hemolysis of a 0.8% suspension of sheep red blood cells) of 100 mg/mL and finally *Atolla wyvillei* was used for lethality tests against shrimp *Palaemon paucidens* with LD_50_ 2 mg/g [70].

### 2.4. Porifera

Marine sponges are the largest source of new marine natural products [71] and have provided a rich array of biologically important compounds [1]. Deep-water species of marine sponges have already provided important anticancer leads such as halicondrin and discodermolide [72] and are a rich new source of biologically and structurally interesting molecules. Four species of Porifera from the deep sea have shown biological activity as reported in Table 4.

Hitora et al. isolated from *Petrosia* sp. six linear acetylenes (−)-duryne and (−)-durynes B–F. These compounds showed cytotoxic activity against HeLa cells with IC_50_ values between 0.08 and 0.50 μM [73]. Desoubzdanne et al. [74] isolated from a deep-sponge *Xestospongia* sp. the molecules alisiaquinones A–D, and found that compound C displayed a submicromolar activity on *Plasmodium falciparum.* From *Leiodermatium* sp. a promising new lead for anticancer drug discovery Leiodermatolide was found to exhibit potent and selective antimitotic activity IC_50_ < 10 nM against a range of human cancer cell lines by inducing G2/M cell cycle arrest [75]. Minkyun Na et al., [76] isolated from a new deep-water Alaskan sponge species of the genus *Latrunculia* two compounds, discorhabdin A and dihydrodiscorhabdin C, that showed anti-hepatitis C virus (HCV) activity with an EC_90_ < 10 μM (Table 4).

### 2.5. Mesopelagic Species

Lauritano et al. [77] studied the potential antimicrobial (Gram-negative bacteria *Escherichia coli* and *Klebsiella pneumoniae*, the Gram-positive bacteria methicillin resistant/sensitive *Staphylococcus aureus*, and *Mycobacterium tubercolosis*) and anticancer activity (A549-lung cell line, A2058-melanoma cell line, HepG2 epatocarcinoma cell line, MCF-7-breast cell line and MiaPaca-2-pancreas cell line) of seven extracts of mesopelagic species (*Meganyctiphanes norvegica; Hygophum benoiti*, *Myctophum punctatum*, *Lampanyctus crocodilus*, *Argyropelecus hemygimnus*, *Chauliodus sloani* and *Stomias boa boa*). They found the most significant activity in the fish *Myctophum punctatum* (Family Mictophidae) and the krill *Meganyctiphanes norvegica* (Family Euphausiidae) (Table 5). *M. punctatum* showed anticancer activity against A549 and MCF-7 cancer cells, while *M. norvegica* was more active against HepG2 cancer cell lines. For *M. punctatum* IC_50_ for A549 was 13.77–23.26, while the IC_50_ for MCF7 was 25.34–29.62 μg/mL. For *M. norvegica* IC_50_ for HepG2 cells was between 3.81 and 7.51 μg/mL. *M. punctatum* and *M. norvegica* extracts were able to inhibit the growth of methicillin-resistant *Staphylococcus aureus* (MRSA) and methicillin-sensitive *Staphylococcus aureus* (MSSA). *M. punctatum* extract was able to inhibit 100% MRSA viability at extract concentrations between 40 and 320 μg/mL. *M. norvegica* was active against MRSA from 80–320 μg/mL [77].

## 3. Other Deep-Sea Organisms

In addition to the above reported organisms, there are other species living in deep environments with biological activities (Table 6). Appleton et al. [78] isolated Rossinones A and B from the colonial sea squirt *Aplidium* sp. (sampling depth at 200 m) that inhibited superoxide production when either *N*-formyl-methionyl-leucyl-phenylananine (fMLP) (IC_50_ 1.9 and 2.5 μM, respectively) or phorbol myristate acetate (PMA) (IC_50_ 0.8 and 0.7 μM, respectively) were used to activate a respiratory burst. These two compounds also showed selective antiviral activity against DNA virus HSV-1 at 2 μg/disk.

From the echinoderm species *Holopus rangii* (sampling depth at 358 m), Wangun et al. [79] isolated gymnochromes E and F, and 7-bromoemodic acid. Gymnochrome E inhibited the proliferation of NCI/ADRRes (multidrug-resistant ovarian cancer cell line) with an IC_50_ value of 3.5 μM. Furthermore, gymnochrome E exhibited minimum inhibitor concentrations (MICs) of 25 μg mL^−1^ against both *Staphylococcus aureus* and methicillin-resistant *S. aureus* (MRSA), while gymnochrome F exhibited MICs of 12.5 μg mL^−1^ against *S. aureus* and MRSA [79]. The deep-sea mollusc *Bathymodiolus thermophilus*, collected from an active hydrothermal vent in the north of Lucky Strike in the Mid-Atlantic Ridge, at a depth of 1733, has furnished the first reported molluscan deep-sea small metabolites, bathymodiolamides A and B, which exhibited apoptosis induction and potential anticancer activity. The compounds inhibit the growth of two cancer cell lines, cervical cancer HeLa cells, with IC_50_ 0.4 μM and 0.5 μM, respectively, and breast cancer MCF-7 cells with IC_50_ values of 0.1 μM and 0.2 μM, respectively [80].

## 4. Conclusions

Since the first report of biologically active MNP spongothymidine in 1950, ~30,000 MNPs have been identified, but only 0.05% of these have reached the market and are currently used for the treatment of human pathologies. In the last 70 years, there has been an increasing interest in MNPs as demonstrated by the increased number of publications (Figure 1). Studies have highlighted that ~70% of MNP have structural scaffolds that are unique for the marine environment. Due to sampling and culturing difficulties, the deep ocean has been poorly studied compared to other marine environments. However, recent molecular tools, in situ experiments, sensor-tagged species and video recordings are giving a boost to the available limited biological knowledge of the deep-sea. Studies have provided evidence that thousands of species live in the deep sea, mainly microorganisms, suggesting that the deep sea may be a potential rich source of structurally diverse, biologically active compounds waiting to be explored. In the deep-sea environment there are a high number of single rare species, with more than half being new to science, with some taxa possessing >95% of undescribed species. The aims of this review were (1) to provide an overview of the bioactive molecules isolated from deep extreme environments already on the market or under investigation for possible industrial applications and (2) to encourage further research to study the potential of deep-sea organisms.

In the last 10 years, several projects have been funded to implement an eco-sustainable approach to drug discovery, favoring species cultivation, advanced dereplication, metabolite production stimulation in the laboratory and in silico analyses to avoid disruptive collection practices in the deep sea. These efforts will also support preservation strategies of deep-sea ecosystems and mitigation of exploitation impacts.

Other approaches are being implemented to study the deep sea, such as genomics, transcriptomics, and metabolomics, combined with innovative chemical analyses, phenotypic screening as well as bioinformatic tools and genetic engineering methodologies. Increased interest in natural product drug discovery in the deep sea over the last decade can be accelerated via academic and biotechnology industry collaboration. A multidisciplinary approach may represent a successful method to identify and biological characterize new marine natural products for the next generation of new bioactive compounds that may be even more potent and selective for the treatment or prevention of human pathologies.

## Figures and Tables

**Table 1:** Bioactive compounds from deep-sea bacteria.

Marine Organism	Compound	Activity/Experimental Model	Depth/Site	Concentrations	Ref.
*Salinispora tropica*	Salinosporamide A	Proteasome inhibitor against NCI-H226-non-small cell lung cancer, SF-539 CNS cancer, SK-MEL-28 skin melanoma, and MDA-MB-435 breast cancer.	1100 m Tropical and subtropical marine sediments	LC_50_ < 10 nM	[23]
*Streptomyces* sp. NTK 935	Benzoxacystol 245	Inhibit recombinant Glycogen synthase kinase 3 beta (GSK-3β)	3814 m Canary basin	IC_50_ = 1.35 ± 0.15 μM	[25]
*Streptomyces* sp. M-207	Lobophorin K	Antiproliferative activity against: MiaPaca-2 and MCF7	1800 m Lophelia pertusa	IC_50_ = 34.0 ± 85.1 and 23.0 ± 8.9 μM, respectively	[26]
*Bacillus subtilis B5*	7, 13-epoxyl-macrolatin A	Inhibitor of LPS-induced inflammatory mediator expression in RAW 264.7 cells	3000 m Pacific Ocean	From 50 to 40 μM	[27]
*Marianactinospora thermotolerans* SCSIO00652	Marinacarbolines A–D 13-N-demethyl-methylpendolmycin Methylpendolmycin-14-*O-*α-glucoside	Antimalarial activity against: *Plasmodium falciparum*	3865 m South China Sea	IC_50_ = (A) 1.92 μM (C) 3.09 μM, (D) 5.39 μM	[28]
*Marinactinospora thermotolerans* SCSIO 00652	Marthiapeptide A	Antimicrobial against: *M. luteus*, *S. aureus*, *B. subtilis*, *B. thuringiensis*	3865 m South China Sea	MIC = 2, 8.4 and 2 μg/mL	[29]
*Marinactinospora thermotolerans* SCSIO 00652	Marthiapeptide A	Cytotoxic activity against a panel of human cancer cell lines	3865 m South China Sea	IC_50_ = ranging from 0.38 to 0.52 μM	[29]
*Streptomyces scopuliridis* SCSIOZJ 46	Desotamide B, C, D	Antimicrobial against: *S. aureus*, *S. pneumoniae*, MRSE shhs-E1	3536 m South China Sea	MIC = 16, 12.5, and 32 μg/mL, respectively	[30]
*Streptomyces drozdowiczii* SCSIO10141	Marfomycins A, B, E	Antimicrobial against: *M. luteus*	1396 m South China Sea	MIC = 0.25, 4, and 4 μg/mL, respectively	[31]
*Streptomyces* sp. SCSIO 01127	Lobophorin F	Antimicrobial against: *S. aureus*, *E. faecalis*	1350 m South China Sea	MIC = 8 μg/mL for both	[32]
*Streptomyces* sp. 12A35	Lobophorin H	Antimicrobial against: *B. subtilis*	2134 m South China Sea	MIC = 3.13 μg/mL	[33]
*Verrucosispora* sp. AB 18-032	Abyssomicin C	Antimicrobial against: MRSA, vancomycin resistant *Enterococcus faecium*, *S. aureus*	289 m Japanese Sea	MIC = 4 μg/mL and 13 μg/mL	[34]
*Streptomyces niveus* SCSIO3406	Marfuraquinocins A, C, D	Antimicrobial against: *S. aureus*, MRSE shhs-E1	3536 m South China Sea	MIC = 8 μg/mL	[35]
*Micromonospora matsumotoense* M-412	Paulomycin G	Antimicrobial against: *E. coli* strains MB5746 and MRSA	2000 m Cantabrian Sea sediments	MIC_90_ = 4.5 and 50 μg/mL, respectively	[36]
*Micromonospora matsumotoense* M-412	Paulomycin G	Antiproliferative against HepG2, MCF7, MiaPaca-2.	2000 m Cantabrian Sea sediments	IC_50_ = 4.30 ± 0.42 μM, 1.58 ± 0.12 μM, 2.70 ± 0.25 μM, respectively	[36]
*Bacillus subtilis*	Lipoamicoumacins A–D, bacilosarcin analogue and six amicoumacins	Cytotoxicity against HeLa cells and antibacterial activity against *B. subtilis*, *S. aureus* and *Laribacter hongkongensis*	1000 m Red Sea	-	[37]
*Dermacoccus abyssi* sp.	Dermacozines F and G	Cytotoxic activity against the leukaemia cell line K562	10,898 m Mariana Trench sediment	IC_50_ values of 9 and 7 μM	[38]
*Pseudonocardia* sp. SCSIO 01299	Pseudonocardians A–C	Cytotoxic activity against SF-268, MCF-7 and NCI-H460	3258 m South China Sea	IC_50_ from 0.01 to 0.21 μM	[39]
*Pseudonocardia* sp. SCSIO 01299	Pseudonocardians A–C	Antibacterial activities against *S. aureus* ATCC 29213, *Enterococcus faecalis* ATCC 29,212 and *Bacillus thuringensis* SCSIO BT01	3258 m South China Sea	MIC values of 1–4 μg mL^−1^	[39]
*Streptomyces lusitanus*	Grincamycin A–E	Cytotoxic activity against HepG2, SW-1990, HeLa, NCI-H460, MCF-7 and B16	3370 m South China Sea	IC_50_ from 1.1 to 31 μM	[40]
*Streptomyces* sp. SCSIO 03032	Spiroindimicins B, C and D	Cytotoxic activity against several cancer cell lines	3412 m Indian Ocean	IC_50_ from 4 to 12 μg mL^−1^ for Spiroindimicin B, IC_50_ from 6 to 15 μg mL^−1^ for Spiroindimicin C, -	[41]
*Streptomyces* sp. NTK 937	Caboxamycin	Inhibitory activity against Gram-positive bacteria, *B. subtilis*, *S. lentus*, *S. epidermidis*, the yeast *Candida glabrata*, the phytopathogenic bacteria *Xanthomonas campestris* and *Ralstonia solanacearum,* and against the opportunistic pathogen *Staphylococcus epidermidis*	3814 m Atlantic Ocean	IC_50_ = 8 μM, 20 μM, 43 μM, 117 μM, 43 μM, 176 μM and 43 μM, respectively	[42]
*Streptomyces* sp. NTK 937	Caboxamycin	Cytotoxic activity towards gastric adenocarcinoma (AGS), HepG2 and MCF-7.	3814 m Atlantic Ocean	IC_50_ = 28.6–29.4 μM	[42]
*Streptomyces* sp. NTK 937	Caboxamycin	Weak inhibitor of bovine brain phosphodiesterase	3814 m Atlantic Ocean	IC_50_ = 148 μM	[42]
*Nocardiopsis alba*	Diketopiperazines	Cytotoxicity against MCF7 and SF-268 human cancer cell lines	1000 m Indian Ocean	IC_50_ = 4.6 and 12.7 μM, respectively	[43]
*Bacillus pumilus* MB 40	Bis (2-ethylhexyl) phthalate (BEHP)	Antiproliferative effect against human erythroleukemic K562 through apoptosis.	1000 m Red Sea	IC_50_ = 21 μM	[44]

Abbreviations are: LC_50_ for lethal concentration; MIC for minimum inhibitory concentration; IC_50_ for the concentration (μg/mL) inducing 50% loss of cell viability; NCI-H226 for non-small cell lung cancer; SF-539 for human glioblastoma cell line; SK-MEL-28 for skin melanoma cell line; MDA-MB-435 breast cancer cell line; HepG2 for Hepatocellular carcinoma; MiaPaca-2 for human pancreatic carcinoma; MCF-7 for breast cancer; “-” means that there is a lack of corresponding information.

**Table 2:** Bioactive compounds from deep-sea fungi.

Marine Organism	Compounds	Activity/Experimental Model	Depth/Site	Concentrations	Ref.
*Aspergillus fischeri* FS452	Fiscpropionates A and C	Inhibitory activities against *Mycobacterium tuberculosis* protein-tyrosine phosphatase B	3000 m Indian Ocean	IC_50_ = 5.1 and 4 μM, respectively	[46]
*Emericella* sp. SCSIO05240	Emerixanthones A and C	Antibacterial activity against *E. coli*, *K. pneumoniae*, *S. aureus*, *E.faecalis*, *A. Baumanni*, *A. hydrophila*	3258 m South China Sea	Diameters of inhibition zones were 4–6 mm	[47]
*Emericella* sp. SCSIO05240	Emerixanthone D	Antifungal activity against *Fusarium* sp., *Penicillium* sp., *Aspergillus niger*, *Rhizoctonia solani*, *Fusariumoxy sporium f*. sp. *niveum*, *Fusariumoxy sporium f*. sp. *cucumeris*	3258 m South China Sea	Diameters of inhibition zones were 3–4 mm	[47]
*Penicillium aculeatum* SD-321	Compound 1 (peniciaculin A) Compounds 2 (peniciaculin B) and 4 (1-hydroxyboivinianin A) Compound 6 (bisabolane)	Antibacterial activity against: *Micrococcus luteus* and *Vibrio alginolyticus* *Edwardsiella tarda* and *Vibrio harveyi* *Staphyloccocus aureus* and *Vibrio parahemolyticus*	2038 m South China Sea	MIC = 1.0 and 2.0 μg/mL, respectively MIC = 8.0 and 4.0 μg/mL, respectively MIC = 0.5 μg/mL	[48]
*Penicillium aculeatum* SD-321	Compound 1 (peniciaculin A) Compounds 7 and 10 (bisabolanes)	Antifungal activity against: *Alternaria brassicae* *Gaeumannomyces graminis*	2038 m South China Sea	MIC = 0.5 μg/mL MIC = 0.5 μg/mL	[48]
*Penicillium* sp. F23-2	Penycyclones A–E	Antimicrobial activity against the Gram-positive bacterium Staphylococcus aureus	5080 m Jiaozhou Bay, Qingdao, China	MIC = 0.3 to 1.0 μg/mL	[49]
*Spiromastix* sp. MCCC3A00308	Spiromastilactones A–M	Antiviral activity against influenza virus replication in vitro	2869 m South Atlantic	Table 3 in the reference	[50]
*Simplicillium oblavatum* EIODSF 020	Simplicilliumtide J, Verlamelins A and B	Antifungal activity against *Aspergillus versicolor* and *Curvularia australiensis*	4571 m East Indian Ocean	MIC = 50 μg/mL	[51]
*Simplicillium oblavatum* EIODSF 020	Simplicilliumtide J, Verlamelins A and B	Antiviral activity against HSV-1	4571 m East Indian Ocean	IC_50_ = 14.0, 16.7 and 15.6 μM/mL, respectively	[51]
*Acremonium* sp.	Acremeremophilanes B and E	Inhibitory effects towards LPS-induced NO production in RAW 264.7	2869 m South Atlantic Ocean	EC_50_ = 8 μM and 15 μM, respectively	[52]
*Eutypella* sp. MCCC3A00281	Eutyperemophilanes I and J	Inhibitory effects towards LPS-induced NO production in RAW 264.7	5610 m South Atlantic Ocean	IC_50_ = 8.6 and 13 μM	[53]
*Penicillium chrysogenum* SCSIO411001	Trans-epoxyamides, chrysamides C	Inhibitory effects on the Interleukin-17 production	3386 m Indian Ocean	1 μM for 40.06% of inhibition	[54]
*Myrothecium* sp. BZO-L062	(−)-1*S*-myrothecol and (+)-1*R*-myrothecol	Anti-inflammatory, inhibiting NO formation in LPS-treated macrophage-like cells	2130 m Yongxing Island	EC_50_ = 1.20 and 1.41 μg/mL	[55]
*Aspergillus* sp. SCSIOW2	Cyclopenol and cyclopenin	Inhibited the LPS-induced formationof nitric oxide and the secretion of IL-6 in RAW 264.7 cells	2439 m South China Sea	IC_50_ = 50–100 μM	[56]
*Penicillium chrysogenum* MCCC 3A00292	Cyclopenol	Inhibition of human Bladder cancer cell line BIU-87	-	IC_50_ = 8.34 μM	[57]
*Penicillium* spp. and *Eurotium* sp.	Fructigenine A, cyclopenol, echinulin, flavoglaucin, and viridicatol	Inhibition of protein-tyrosine phosphatase 1B	- Wan Island, Korea	IC_50_ = 10.7, 30.0, 29.4, 13.4, and 64.0 μM, respectively	[58]
*Graphostroma* sp. MCC3A00421	Reticulol	Decrease the rates of degranulation and histamine release in RBL-2H3 cells	2721 m Atlantic hydrotermal sulfide	IC_50_ =13.5 and 13.7 μM	[59]
*Penicillum granulatum* MCCC 3A00475	Spirograterpene A	Antiallergic activity	2284 m Prydz Bay of Antarctica	20 μg/mL	[60]
*Penicillium chrysogenum* SCSIO 07007	Chrysopyrones A and B	Inhibited protein-tyrosine phosphatase-1B (PTP1B) involved in diabetes mellitus	1000 m Western Atlantic	IC_50_ = 9.32 and 27.8 μg/mL, respectively	[61]
*Engyodontium album* DFFSCS021	Engyodontiumone H (Compound 8) and a polyketide (Compound 16) Compounds 8 and the polyketides compounds 15 and 16	Cytotoxic activity against human histiocitic lumphoma U937 Antibacterial activity against *Escherichia coli* and *Bacillus subtilis*	3739 m South China Sea	IC_50_ = 4.9 and 8.8 μM, respectively MIC ≤ 64 μg/mL	[62]
*Acrostalagmus luteoalbus* SCSIO F457	Luteoalbusins A, B	Cytotoxic activity against SF-268, MCF-7, NCI-H460 and HepG2	2801 m South China Sea	IC_50_ from 0.23 to 1.31 μM	[63]
*Aspergillus versicolor*	Oxisterigmatocystin A–C	Cytotoxic activity against A549 and HL-60	800 m Pacific Ocean	Moderate low micromolar cytotoxicity	[64]
*Penicillium* sp. MCCC 3A00005	Brevione I	Cytotoxicity against MCF-7 cells and A549 cells	5115 m East Pacific	IC_50_ = 7.44 μM and 32.5 μM, respectively	[65]
*Penicillium* sp. MCCC 3A00005	Brevione A	Cytotoxicity against MCF-7 cells	5115 m East Pacific	IC_50_ = 28.4 μM	[65]
*Penicillium* sp. F00120	Penicilliumin A	Moderately inhibited the in vitro proliferation of mouse melanoma (B16), human melanoma (A375), and human cervical carcinoma (Hela) cell lines	1300 m South China Sea	GI_50 =_ 27.37, 22.88, and 44.05 μg/mL, respectively	[66]
*Penicillium* sp. F23-2	Sorbicillamines A–E	Cytotoxic activity on HeLa, BEL-7402, HEK-293, HCT-116, and P388 cell line	5080 m Huiquan bay, yellow Sea	IC_50_ > 10 μM	[67]

Abbreviations: EC_50_ for Half-maximal effective concentration; MIC for minimum inhibitory concentration; IC_50_ for the concentration (μg/mL) inducing 50% loss of cell viability; GI_50_ for 50% of maximal inhibition of cell proliferation; MCF7 for breast cancer cell line, SF-268 for human glioblastoma cell line, LPS for lipopolysaccharide; NO for nitric oxide; RAW 264.7 for macrophage cell line; RBL-2H3 for basophilic leukemia cell line; A549 for lung cancer cells; HeLa for cervical cancer cells; BEL-7402 for human papillomavirus-related endocervical adenocarcinoma; HEK-293 for human embryonic kidney; HCT-116 for human colon cancer cells; P388 for murine leukemia cells lines; “-“ means that there is a lack of corresponding information.

**Table 3:** Bioactive compounds from deep-sea Cnidaria.

Marine Organism	Activity/Experimental Model	Depth/Site	Concentrations	Ref.
*Aegina citrea*	Citotoxicity test against Leukemia L1210 cells	200–1000 m Sagami Bay	IC_50_ = 100 mg/mL	[70]
*Crossota rufobrunea*	Hemolytic activity test of suspension of sheep red blood cells	200–1000 m Sagami Bay	ED_50_ = 100 mg/mL	[70]
*Atolla wyvillei*	Crustacean letality test against shrimp *Palaemon paucidens*	200–1000 m Sagami Bay	LD_50_ = 2 mg/g	[70]

Abbreviations: IC_50_ for the concentration (μg/mL) inducing 50% loss of cell viability; ED_50_ for effective dose for 50% hemolysis of a 0.8% suspension of sheep red blood cells; LD_50_ for lethal activity; HSV1 for herpes simplex 1.

**Table 4:** Bioactive compounds from deep-sea Porifera.

Marine Organism	Compound	Activity/Experimental Model	Depth/Site	Concentration	Ref.
*Petrosia* sp.	(−)-Duryne and (−)-Durynes B–F	Cytotoxic against HeLa cells	415 m Japan	IC_50_ from 0.08 to 0.5 μM	[73]
*Xestospongia* sp.	Alisiaquinones A–C	Antimalarial against *Plasmodium falciparum*	250/400 m Australia	Submicromolar	[74]
*Leiodermatium* sp.	Leiodermatolide	Antimitotic	401 m USA	IC_50_ < 10 nM	[75]
*Latrunculia* sp.	Discorhabdin A and Dihydrodiscorhabdin C	Anti-HCV	230 m Alaska	EC_90_ < 10 μM	[76]

Abbreviations: IC_50_ for the concentration (μg/mL) inducing 50% loss of cell viability; EC_90_ for the 90% effective concentration in the HCV replicon assay; less than 10 μM is considered active. HCV for hepatitis C virus. HeLa cervical cancer cell lines.

**Table 5:** Bioactive compounds from deep-sea mesopelagic organisms.

Marine Organism	Activity/Experimental Model	Depth/Site	Concentrations	Ref.
*Myctophum punctatum*	Antiproliferative activity against A549 and MCF7	200–1000 m Straits of Messina (central Mediterranean Sea)	IC_50_ = 13.77–23.26 and 25.34–29.62 μg/mL, respectively	[77]
	Antibacterial activity against MRSA		IC_50_ = 40 and 320 μg/mL	
*Meganyctiphanes norvegica*	Antiproliferative activity against HepG2	200–1000 m Straits of Messina (central Mediterranean Sea)	IC_50_ = 3.81 and 7.51 μg/mL	[77]
	Antibacterial activity against MRSA		IC_50_ = 80–320 μg/mL	

Abbreviations: IC_50_ for the concentration (μg/mL) inducing 50% loss of cell viability; MCF7 for breast cancer cell line; A549 for lung cancer cells; MRSA for methicillin resistant *Staphylococcus aureus*; MSSA for methicillin sensitive *Staphylococcus aureus*.

**Table 6:** Synopsis of new natural products isolated from deep-sea organisms.

Phylum	Marine Organism	Compound	Activity/Experimental Model	Depth/Site	Concentration	Ref.
Chordata	*Aplidium* sp.	Rossinones A and B	Antileukemic, anti-inflammatory and antiviral against HSV-1	200 m Antarctica	IC_50_ = 1.9 and 2.5 μM 2 μg/disk	[78]
Echinodermata	*Holopus rangii*	Gymnochromes E and F	Antiproliferative against NCI/ADRRes	358 m Caribbean	IC_50_ = 3.5 μM	[79]
Mollusca	*Bathymodiolus thermophilus*	Bathymodiolamides A and B	Anticancer against HeLa and MCF-7 cell lines	1733 m Mid-Atlantic ridge	IC_50_ = 0.4 μM and 0.5 μM; IC_50_ = 0.1 μM and 0.2 μM	[80]

Abbreviations: IC_50_ for the concentration (μg/mL) inducing 50% loss of cell viability. NCI/ADRRes multidrug-resistant ovarian cancer cell line; HSV-1 for herpes simplex-1. HeLa for cervical cancer cells, MCF7 for breast cancer cell.

## Data Availability

Not applicable.
